# Editorial: New opportunities for diagnosis and control of Chagas Disease to reach the 2030 goals for elimination

**DOI:** 10.3389/fpara.2023.1258999

**Published:** 2023-07-27

**Authors:** María V. Periago, Roberto Chuit

**Affiliations:** ^1^ Consejo Nacional de Investigaciones Científica y Técnicas (CONICET), Fundación Mundo Sano, Buenos Aires, Argentina; ^2^ Académica Nacional de Medicina, Institute of Epidemiological Research (IIE), Buenos Aires, Argentina

**Keywords:** Chagas acute and chronic phases, Chagas cardiomyopathy, Chagas, diagnosis, elimination

The World Health Organization´s (WHO) roadmap for neglected tropical diseases (NTDs), launched in 2021, details the global targets to prevent, control, eliminate or eradicate this group of diseases by 2030 (WHO, 2020). The main target for Chagas Disease (ChD), caused by the parasite *Trypanosoma cruzi*, is elimination as a public health problem, given the zoonotic nature of this disease, with active sylvatic cycles (de Arias et al., 2022). The specific target set out for ChD is broad and based on two indicators: 1) the number of countries achieving interruption through all the parasites´ transmission routes, and 2) antiparasitic treatment coverage of 75% of the eligible population (WHO, 2020). According to the roadmap, both must be achieved.

Unfortunately, many individuals at risk for infection in endemic countries or those infected who have moved to non-endemic countries do not have adequate access to diagnosis, and even fewer have access to specific treatment. Given the current mobilization of populations from rural to urban sites or even from one country to another, associated with transmission routes of the parasite to humans, which do not require a vector, make it necessary to develop tools tailored to the different situations that arise, to achieve the goals established in the road map. We must rethink how this new reality of the disease impacts the organization of the health system, accessibility, communication, and culture, among others.

One of the critical issues is granting access to diagnosis using validated techniques that can be adapted to different situations. Lopez-Albizu et al. (2023) review the available techniques for laboratory diagnosis of *T. cruzi*, either during the acute or chronic phase, focusing on those available in Latin America. The authors point out that although there has been progress in the availability, evaluation, and validation of new technologies for early detection of *T. cruzi* infection, tools to allow access to diagnosis at the primary healthcare level are still lacking, despite advancement in other infections such as H1N1 influenza virus or SARS-CoV-2 (Lopez-Albizu et al. 2023). Additionally, even when an individual is diagnosed, management of patients with ChD, either in the acute or chronic phase, is not always adequate, given a lack of knowledge from medical personnel (Pereiro et al., 2018; Echeverría et al., 2020). In this Research Topic, Chuit et al. (2023) focus on managing patients with Chagasic Cardiomyopathy (ChD-MCP) in Argentina, using historical medical records from different institutions. Through analysis of the records, the authors could show the most common risk factors in patients with ChD-MCP and the specific studies performed on the patients, among others (Chuit et al. 2023). Most importantly, this study detected and corroborated that many patients were not adequately studied.

Both these studies (Chuit et al., 2023; Lopez-Albizu et al., 2023) highlight some of the challenges to achieving global objectives for reaching the 2030 goals for the elimination of ChD as a public health program, while the other two articles published as part of this Research Topic focus on the need to develop programs that use integral approaches (Gold and Hermida, 2023) and intersectoral partnerships (Abril, 2023). Gold and Hermida, (2023) mention the importance of the social determinants of health that need to be considered when implementing strategies to prevent and control ChD, an NTD that is intimately tied to poverty and migration. The authors showcase a program that was implemented in the endemic area of northwestern Argentina and detail some of the characteristics that aided its sustainability: consideration of socio-environmental factors, community participation and engagement, adaptive methodology, and joint work with local authorities (Gold and Hermida, 2023). Therefore, it´s essential to have a multidisciplinary team in these types of programs and, as stated by Abril, (2023), intersectoral partnerships for collaboration. In this opinion piece, Abril, 2023 summarizes the horizontal cooperation programs that have been implemented in the past and which were not enough to reach the goals set by WHO to control the disease while detailing exciting new collaborations and initiatives paving the road to 2030 (Abril, 2023).

Many advances have been achieved in reducing vector transmission of *T. cruzi* in the endemic region of the Americas (PAHO/WHO, 2018). New modes of transmission, i.e., mother-to-child transmission (MTCT), are now becoming the most crucial cause in endemic and non-endemic countries due to migratory processes from rural areas to cities within a country and from endemic to non-endemic countries (Carlier et al., 2019; Pinazo et al., 2020). To specifically tackle this matter, the Pan American Health Organization (PAHO) has launched a comprehensive framework for the elimination of MTCT of HIV, syphilis, Hepatitis B, and Chagas (EMTCT-Plus) (PAHO, 2017). Fortunately, the tools and means to reach the 2030 goals and eliminate ChD as public health problem are currently available; we now need to learn from past experiences and look forward to working together and ensuring that they are made available to those who need them.

## Author contributions

All authors listed have made a substantial, direct, and intellectual contribution to the work and approved it for publication.
